# A randomised phase II trial of preoperative chemotherapy of cisplatin–docetaxel or docetaxel alone for clinical stage IB/II non-small-cell lung cancer: results of a Japan Clinical Oncology Group trial (JCOG 0204)

**DOI:** 10.1038/sj.bjc.6604613

**Published:** 2008-08-26

**Authors:** H Kunitoh, H Kato, M Tsuboi, H Asamura, H Tada, K Nagai, T Mitsudomi, T Koike, K Nakagawa, Y Ichinose, M Okada, T Shibata, N Saijo, JCOG Lung Cancer Surgical Study Group, Tokyo, Japan

**Affiliations:** 1 Department of Medical Oncology, National Cancer Center Hospital, 5-1-1 Tsukiji, Chuo-ku, Tokyo 104-0045, Japan; 2 Department of Thoracic Surgery, Tokyo Medical University, 6-7-1 Nishishinnjuku, Shinjuku-ku, Tokyo 160-0023, Japan; 3 Division of Thoracic Surgery, National Cancer Center Hospital, 5-1-1 Tsukiji, Chuo-ku, Tokyo 104-0045, Japan; 4 Department of General Thoracic Surgery, Osaka City General Hospital, 2-13-22 Miyakojima-Hondori, Miyakojima-ku, Osaka 534-0021, Japan; 5 Department of Thoracic Surgery, National Cancer Center Hospital East, 6-5-1 Kashiwanoha, Kashiwashi, Chiba 277-8577, Japan; 6 Department of Thoracic Surgery, Aichi Cancer Center, 1-1 Kanokoden, Chikusa-ku, Nagoya City, Aichi 464-8681, Japan; 7 Department of Thoracic Surgery, Niigata Cancer Center, 2-15-3, Kawagishi-cho, Niigata-shi, Niigata 951-8566, Japan; 8 Thoracic Oncology Center, Cancer Institute Hospital, 3-10-6 Ariake, Koto-ku, Tokyo 135-8550, Japan; 9 Department of Thoracic Oncology, National Kyushu Cancer Center, 3-1-1 Notame, Minami-ku, Fukuoka City, Fukuoka 811-1395, Japan; 10 Department of Surgical Oncology, Hiroshima University Research Institute for Radiation Biology and Medicine, 1-2-3 Kasumi, Minami-ku, Hiroshima City, Hiroshima 734-8553, Japan; 11 JCOG Data Center, Center for Cancer Control and Information Services, National Cancer Center, 5-1-1 Tsukiji, Chuo-ku, Tokyo 104-0045, Japan; 12 National Cancer Center Hospital East, 6-5-1 Kashiwanohara, Kashiwashi, Chiba 277-8577, Japan

**Keywords:** cisplatin, docetaxel, lung cancer, non-small cell, preoperative chemotherapy, stage IB/II

## Abstract

Preoperative chemotherapy is a promising strategy in patients with early-stage resectable non-small-cell lung cancer (NSCLC); optimal chemotherapy remains unclear. Clinical (c-) stage IB/II NSCLC patients were randomised to receive either two cycles of docetaxel (D)–cisplatin (P) combination chemotherapy (D 60 mg m^−2^ and P 80 mg m^−2^ on day 1) every 3–4 weeks or three cycles of D monotherapy (70 mg m^−2^) every 3weeks. Thoracotomy was performed 4–5 weeks (DP) or 3–4 weeks (D) after chemotherapy. The primary end point was 1-year disease-free survival (DFS). From October 2002 to November 2003, 80 patients were randomised. Chemotherapy toxicities were mainly haematologic and well tolerated. There were two early postoperative deaths with DP (one intraoperative bleeding and one empyema). Pathologic complete response was observed in two DP patients. Docetaxel–cisplatin was superior to D in terms of response rate (45 *vs* 15%) and complete resection rate (95 *vs* 87%). Both DFS and overall survival were better in DP. Disease-free survival at 1, 2 and 4 years were 78, 65 and 57% with DP, and were 62, 44 and 36% with D, respectively. Preoperative DP was associated with encouraging resection rate and DFS data, and phase III trials for c-stage IB/II NSCLC are warranted.

Surgery is the standard of care for clinical (c-) stage IB/II non-small-cell lung cancer (NSCLC), but the treatment outcome remains poor, with 5-year survival rates of 50% or less (Mountain, 1997; Goya *et al*, 2005). The majority of post-surgical relapse occurs as distant metastases (Pisters and Le Chevalier, 2005); therefore, effective systemic therapy is necessary. Recently, a series of postoperative adjuvant chemotherapy trials reported modest but significant improvement in survival, mainly in patients with pathological stage II or IIIA NSCLC (Arriagada *et al*, 2004; Scagliotti, 2005; Winton *et al*, 2005; Douillard *et al*, 2006). Compliance to the chemotherapy remains a problem (Arriagada *et al*, 2004; Scagliotti, 2005; Winton *et al*, 2005; Douillard *et al*, 2006).

On the other hand, previous small phase III trials had reported that preoperative chemotherapy was better than surgery alone in stage III NSCLC (Rosell *et al*, 1994; Roth *et al*, 1994). Recent trials of preoperative platinum-based chemotherapy have reported promising results in c-stage IB/II NSCLC (Pisters *et al*, 2000; Depierre *et al*, 2002; Rosell *et al*, 2002). One advantage of the preoperative chemotherapy is better tolerability and compliance.

No data are available, however, as to the optimal preoperative therapy strategy for early-stage NSCLC. Although platinum-based ‘standard’ combination chemotherapy regimens have widely been used and reported to be generally safe, results of randomised trials reported nonsignificant but modest excess of post-surgical morbidity and mortality (Depierre *et al*, 2002; Pisters *et al*, 2007). Monotherapy with an active agent is associated with lower response rate, but less toxicity (Delbaldo *et al*, 2004); it might well be favourable for preoperative therapy in early stage, when surgery must not be compromised by adjuvant therapy.

Docetaxel (D) is a semisynthetic taxoid derived from the European yew *Taxus baccata*. It is active against NSCLC either in monotherapy (D)(Fossella *et al*, 1994; Francis *et al*, 1994; Kunitoh *et al*, 1996) or in combination with cisplatin (DP) (Zalcberg *et al*, 1998; Fossella *et al*, 2003). In advanced NSCLC, DP was reported to be better than P–vinca combination (Fossella *et al*, 2003; Kubota *et al*, 2004), one of the ‘standard’ adjuvant therapies. The DP combination was also reported to be active and promising as preoperative chemotherapy in c-stage III NSCLC (Betticher *et al*, 2006).

Docetaxel monotherapy, on the other hand, was reported to be not inferior to DP, with better tolerability in advanced NSCLC (Georgoulias *et al*, 2004). For stage III NSCLC, Mattson *et al* (2003) reported the results of D as preoperative chemotherapy; it was active, and did not compromise surgery.

On the basis of this rationale, we undertook a randomised phase II trial of DP *vs* D in resectable, c-stage IB/II NSCLC. The objectives of the study were to evaluate the safety and efficacy of the preoperative chemotherapy and to select promising one for future phase III trials. The primary end point was the disease-free survival (DFS) rate at 1 year.

## Patients and methods

### Patient eligibility criteria

Patients with untreated, histologically or cytologically documented NSCLC with clinical stage IB (c-T2N0M0), IIA (c-T1N1M0) or IIB (c-T2N1M0 or T3N0M0) were eligible for study entry. Each patient was required to meet the following criteria: 20–74 years of age, Eastern Cooperative Oncology Group (ECOG) performance status (PS) of 0 or 1; measurable disease; and adequate organ function (leukocyte count ⩾4000/*μ*l and ⩽12 000/*μ*l, neutrophil count ⩾2000/*μ*l, platelet count ⩾10^5^/*μ*l, haemoglobin ⩾10.0 g dl^−1^, serum creatinine ⩽the upper limit of the institutional normal range (ULN), creatinine clearance calculated by the Cockcroft–Gault formula ⩾60 ml min^−1^, serum bilirubin ⩽ULN, serum ALT and AST ⩽2 × ULN and PaO_2_ ⩾70 mm Hg). Women who were pregnant or lactating were excluded from the study. Other exclusion criteria included patients with active infection, unstable angina or a history of myocardial infarction within 6 months, interstitial pneumonia or active lung fibrosis, uncontrolled diabetes or hypertension, systemic use of corticosteroid or active concomitant malignancy. Patients with tumour invading the first rib or more superior chest wall (Pancoast type) were also excluded. All mediastinal nodes measuring 1.0 cm or more in size on computed tomographic (CT) scans were required to be biopsied to be histologically benign before patient entry.

Patient eligibility was confirmed by the Japan Clinical Oncology Group Data Centre before registration. The study protocol was approved by the institutional review boards at each participating centre, and all patients provided written informed consent.

### Treatment plan

This was an open-label, randomised trial. Patients were randomly assigned to one of two treatment arms. Dosages of the chemotherapy were based on the regulatory notes and clinical data in Japan (Kubota *et al*, 2004). In the DP combination arm, patients received D at 60 mg m^−2^ as a 1-h intravenous infusion followed by P at 80 mg m^−2^ as a 2-h infusion on day 1. Two cycles of the chemotherapy were repeated at an interval of 4 weeks. The interval was permitted to be shortened to 3 weeks, if the patient was judged to have adequately recovered enough from the first cycle. Surgery (lobectomy or pneumonectomy with systematic lymph node dissection) was performed 4–5 weeks after completion or early termination of the chemotherapy. Patients in the D monotherapy arm received D at 70 mg m^−2^ as a 1-h intravenous infusion on day 1. Three cycles of the chemotherapy were repeated at 3 weeks intervals. Surgery in the D arm was performed 3–4 weeks after completion or early termination of chemotherapy. The preoperative periods were thus set at 8–10 weeks in each arm, which was designed to be easier to accept for the patients and the surgeons.

In each arm, when chemotherapy was judged to be ineffective with ⩾10% unidirectional tumour growth, or when the patient experienced unacceptable toxicity (such as, grade 3 neurotoxicity, grade 2 pulmonary toxicity, grade 3 cardiac toxicity or other grade 4 non-haematological toxicities), chemotherapy was discontinued and the patient was taken up for surgery as clinically indicated. With minor toxicities, such as uncomplicated grade 4 haematologic or grade 3 non-critical, non-haematological toxicities, dosages of subsequent chemotherapy courses were reduced (P by 20 mg m^−2^ and D by 10 mg m^−2^).

No protocol therapy was predetermined for those with unresectable tumours, either during chemotherapy or at operation, and those with microscopically or macroscopically incompletely resected tumours. Those who underwent curative resection were observed until recurrence without additional therapy.

Chemotherapy was supported with routine premedication for hypersensitivity and antiemetics. For the DP arm, ample hydration was ensured. Recombinant human granulocyte colony-stimulating factor was administered when grade 4 neutropaenia or neutropaenic fever occurred.

### Patient evaluation and follow-up

Before study enrolment, a complete medical history and physical examination, blood cell count determinations, biochemistry testing, chest X-ray, ECG, CT scan of the chest and CT scan or ultrasound of upper abdomen were conducted for each patient. Whole-brain CT or magnetic resonance imaging (MRI) or isotope bone scanning was performed if clinically indicated. Positron emission tomography (PET) was not widely available in Japan at the time of the protocol activation and was not routinely used for staging. Blood cell counts, differential WBC counts and biochemistry testing were performed weekly during each course of chemotherapy.

Toxicity of the chemotherapy was evaluated with the National Cancer Institute Common Toxicity Criteria Tumour (NCI-CTC; version 2.0). Tumour responses were assessed radiographically according to the RECIST guideline (Therasse *et al*, 2000). Response confirmation at 4 weeks or longer intervals was not necessitated. Response was assessed by the attending physicians in each participating institution, and no central confirmation was performed. Chest X-ray was taken at each course, and when suggested for even minor tumour growth (⩾10%), confirmatory chest CT was performed to decide on the continuation of chemotherapy.

After curative resection, the patients were followed up with periodic reevaluation. This included chest CT every 6 months for the first 2 years and annually thereafter, until 5 years or tumour recurrence.

### Statistical considerations

This trial was designed as a randomised phase II selection design. Therefore, formal statistical hypothesis testing of the differences between the arms, including the calculation of *P*-values, was not to be performed. The aim was to select the ‘preferable’ preoperative chemotherapy arm for a future definitive phase III trial, with the DFS rate at 1 year as primary end point. The DFS was calculated from the date of enrolment by the Kaplan–Meier method, as was the overall survival (OS).The ‘events’ for the determination of the DFS included tumour relapse after curative surgery, death from any cause and non-curative operation. Those with non-curative operation include patients without surgery and those with incomplete resection, either microscopically or macroscopically. Non-curative operation was to be counted as an event on the date of registration, not on that of surgery. The sample size was determined to provide sufficient probability to choose the ‘preferable’ arm (Simon *et al*, 1985). Assuming DFS rates at 1 year of 70 and 80%, 40 patients per arm were required to correctly select the arm that is not inferior with the probability of 84.9%. The ‘minimal’ DFS rate of 70% was assumed with the prior report from North America, in which the 1- and 2-year survival rates were reported to be 85 and 56%, respectively (Pisters *et al*, 2000). The assumption was rough and might well be inaccurate, for no DFS data were available from the literature. The randomisation was carried out by the JCOG data centre using a minimisation method with c-stage (IB *vs* II) and institutions as balancing factors.

The secondary end points included the objective tumour response to chemotherapy, complete resection rate, intra- and post-surgical morbidity/mortality and the OS rate. Tumour responses in both arms were compared using Fisher's exact test.

During the accrual period, an interim analysis was planned after 40 patients were randomised and followed up for at least 4 months. All analyses were performed with the SAS software version 9.1 (SAS Institute, Cary, NC, USA).

## Results

### Patient characteristics

From October 2002 to October 2003, 80 patients from 18 institutions were enroled and randomised. After 40 patients were randomised, an interim analysis was carried out. Following the JCOG Data and Safety Monitoring Committee's review, the study was continued. One patient in the D arm was found to be ineligible because of the wrong histology (sarcoma). All 80 patients were analysed for characteristics and chemotherapy toxicity, and the 79 eligible patients were analysed for the clinical and pathological response to chemotherapy, surgical results, DFS and OS.

Table 1 lists the characteristics of the patients, which were well balanced between the arms.

### Chemotherapy delivery and toxicity

Table 2 summarises the chemotherapy delivery, and Table 3 summarises toxicity in the subject group. Only 60% in the D arm completed the planned chemotherapy courses, mainly arising from the clinical ineffectiveness of the therapy. On the other hand, compliance was very good in the DP arm, and the toxicity was not greater. Hyponatraemia, probably due to hydration with P administration, was an unexpected toxicity in the DP arm, but it was clinically silent and transient in all the cases. All patients recovered without any particular management, with no clinically relevant sequelae. Other toxicities were mainly haematologic, and both chemotherapy arms were generally well tolerated by the patients.

### Clinical response and pathological results

Table 4 shows the clinical responses to the chemotherapy. The overall response rates, 45% in the DP arm and 15% in the D arm, were compatible with earlier reports for each of the chemotherapy regimen in patients with NSCLC.

Thoracotomy was performed in 39 of the 40 patients in the DP arm, and in 37 of the 39 patients in the D arm. The tumour was surgically resected in 39 (98%) patients in the DP arm, including pneumonectomy in 3 cases, bi-lobectomy in 2 cases and lobectomy in 34 cases. Tumour resection was performed in 35 (90%) patients of the D arm, including pneumonectomy in 1 case, bi-lobectomy in 4 cases and lobectomy in 30 cases. Five patients, including four in the DP arm and one in the D arm, suffered from massive (⩾1 l) intraoperative bleeding: due to severe adhesion in three cases (two in DP and one in D arm), to incomplete suture of the autostapler resulting in injury of pulmonary artery in one case (DP arm) and accidental injury to the aorta in one case (DP arm). None was judged to be related to preoperative therapy. The postoperative complications included one patient with empyema and another with pulmonary oedema, both in the DP arm. There were two surgical deaths, both in the DP arm; one died on postoperative day 59 because of empyema, and another on postoperative day 2 because of massive intraoperative bleeding resulting from surgical injury to the aorta.

Pathological complete resection (R0), without residual tumour found either macroscopically or microscopically, was achieved in 38 (95%) cases in the DP arm, and 34 (87%) cases in the D arm. On pathological examination, 23% of the 75 patients who underwent surgery were found to have N2 or N3 status. Pathologic CR was achieved in two patients, both in the DP arm. Clinical N-stage was poorly correlated to pathological nodal status (Table 5).

### DFS and OS

**Figure 1:**
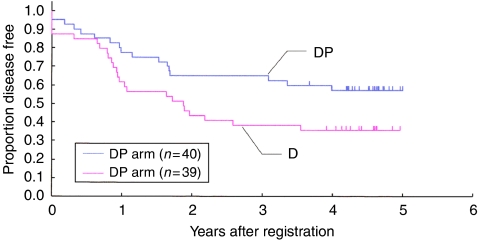
Disease-free survival.

The DFS and OS were updated in November 2007. The DFS rates at 1, 2 and 4 years were 78, 65 and 57% in the DP arm, and were 62, 44 and 36% in the D arm, respectively (Figure 1). Table 6 summarises the outcome at 1 year, the primary end point of the study. The DFS rate at 1 year was 78% (31 out of 40) in the DP arm, which was consistent with the study assumption that it would be 80% in the ‘better’ arm, whereas it was a disappointing 62% (24 out of 39) in the D arm. The 16% difference was more than presumed in the protocol.

**Figure 2:**
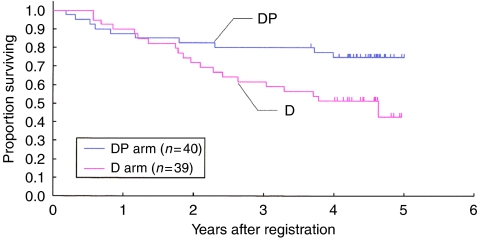
Overall survival.

The OS rates at 1, 2 and 4 years were 88, 83 and 75% in the DP arm, and were 87, 72 and 57% in the D arm, respectively (Figure 2). Both the DFS and the OS rates were better in the DP arm. The OS was better in the DP arm in both adenocarcinoma and non-adenocarcinoma histological subtypes.

## Discussion

As compared with post-surgical adjuvant therapy, preoperative chemotherapy has several practical as well as theoretical advantages (Pisters *et al*, 2000; Pisters, 2003). The practical advantages include better patient tolerance and clinical visualisation of chemotherapy effect.

There are very few reports as to the optimal preoperative therapy strategy. The majority of trials have used ‘standard’ platinum-based doublets (Pisters, 2003). Although they are the ‘standard’ for advanced, stage IV NSCLC, a trade-off between the cytotoxic effect and toxicity of the chemotherapy, not only toxicity itself but also its influence on surgery and post-surgical morbidity and mortality (Depierre *et al*, 2002; Pisters *et al*, 2007), must be considered for preoperative therapy.

In this randomised phase II study, we evaluated DP combination chemotherapy and D monotherapy as preoperative treatment for early stage NSCLC. Although the DFS assumptions of the protocol, 70 *vs* 80% at 1 year, were rough and arbitrary due to lack of historical data, subsequent S9900 trial (Pisters *et al*, 2007) showed DFS rate of 68% in the surgery alone group and 69% in those with preoperative carboplatin–paclitaxel therapy, consistent with our assumption.

Our results showed that single-agent D was inadequate in this setting; an unexpectedly high progression rate led to an early chemotherapy termination rate of as high as 40%. The reason for the high PD rate is unknown. In addition, we tried to minimise the disadvantage of continuation of ineffective chemotherapy by defining the ineffectiveness as ⩾10% tumour size increase instead of ⩾20% in the RECIST guideline (Therasse *et al*, 2000). This subtle decision rule might require centralised confirmation. The DFS rate in the D arm was disappointing and was, in fact, very similar to that in the surgery-alone arm in the S9900 study in the United States (Pisters *et al*, 2007).

On the other hand, both the DFS and OS rates of the DP arm were promising. Disease-free survival at 1 year of 78% was fully consistent with the estimation in the study protocol. Although our data do not refute other platinum-based chemotherapy as candidates of preoperative treatment, it would be justified to conclude that DP was active and promising, regardless of disappointing data of D monotherapy. One might argue that DFS at 1 year was too premature as an end point. Because the DFS and OS curves of the DP arm seem to have reached to plateau at 2 years, DFS at 2 years might be a more appropriate end point.

The number of chemotherapy courses of the DP combination was two, whereas many previous studies used three courses. In the North American trials with carboplatin and paclitaxel, three preoperative courses appeared to have no advantage when compared with two courses (Pisters *et al*, 2000; Pisters, 2003). Although patients with ‘two preoperative courses’ were to have two additional courses after the operation, compliance to the post-surgical courses was very poor anyway (Pisters *et al*, 2000). But, as the majority of the patients appeared fit enough after two courses of DP and a major operation, we could consider the addition of a couple of postoperative chemotherapy cycles at least for responders.

One of the major disadvantages of preoperative therapy is the inaccuracy of the clinical staging, as reported by Depierre *et al* (2002). In our trial, 23% of the 74 patients who underwent thoracotomy were found to have p-N2/N3 disease. In Japan, mediastinoscopy for patients with mediastinal nodes measuring 1 cm or less in size on CT is not performed as a routine clinical practise, and nor was it in our study. Although the introduction of PET may improve the accuracy of the clinical staging, it would still be unlikely to be comparable to surgical staging (Lardinois *et al*, 2003; Cerfolio *et al*, 2004; Shim *et al*, 2005). This would inevitably lead to heterogeneity of the patient population, necessitating a sophisticated study design and large sample size for any future trial on preoperative therapy.

We conclude that the DP combination regimen is active and well tolerated as preoperative chemotherapy, with highly promising survival data. Future clinical trials are warranted based on our results.

## Figures and Tables

**Table 1:** Patient characteristics

**Arm**	**Cisplatin–docetaxel**	**Docetaxel alone**
*N*	40	40
*Clinical stage*		
IB	22	23
II	18	17
		
*Clinical T stage*		
T1	5	5
T2	31	29
T3	4	6
		
*Clinical N stage*		
N0	26	28
N1	14	12
		
*ECOG performance status*		
PS0	35	31
PS1	5	9
		
*Histology*		
Adenocarcinoma	30	24
Squamous cell carcinoma	10	11
Others	0	5
		
*Body weight loss within 6 months*		
None	24	22
⩽5 kg	13	14
>5 kg	1	2
Missing	2	2
		
*Smoking*		
Median smoking	40 pack-years	40 pack-years
Never-smoker	6	8

**Table 2:** Delivery of chemotherapy

**Arm**	**Cisplatin–docetaxel**	**Docetaxel alone**
*N*	40	40
*Completed*	38 (95%)	24 (60%)
*Not completed*	2	16
Ineffective^a^	1	11
Adverse event	1	3
Patient refusal	0	1
Found ineligible	0	1

a Ineffectiveness was judged upon ⩾10% unidirectional increase in tumour size, and did not necessarily mean progressive disease by RECIST.

**Table 3:** Toxicity of chemotherapy

**Arm**	**Cisplatin–docetaxel**	**Docetaxel alone**
*N*	40	40
Grade	2/3/4 (% grade 3+4)	2/3/4 (% grade 3+4)
*Haematological*		
Leukopaenia	18/14/1 (38)	12/15/2 (43)
Neutropaenia	5/16/17 (83)	5/10/21 (78)
Anaemia	4/0/0 (0)	7/0/0 (0)
Thrombocytopenia	1/0/0 (0)	0/0/0 (0)
*Nonhaematological*		
Total bilirubin	4/0/0 (0)	0/0/0 (0)
Serum AST	0/0/0 (0)	3/1/0 (3)
Serum ALT	5/0/0 (0	5/1/0 (3)
Serum creatinine	3/0/0 (0)	0/0/0 (0)
Hypoxia	0/0/0 (0)	3/0/0 (0)
Hypercalcaemia	0/0/0 (0)	0/1/0 (3)
Hyponatraemia	−/6/0 (15)	−/1/0 (3)
Hypersensitivity	0/0/0 (0)	0/1/0 (3)
Fatigue	3/1/0 (3)	0/0/0 (0)
Constipation	4/1/0 (3)	5/0/0 (0)
Diarrhea	3/3/0 (8)	2/0/0 (0)
Nausea	9/7/− (18)	0/0/− (0)
Vomiting	5/1/0 (3)	0/0/0 (0)
Febrile neutropaenia	−/1/0 (3)	−/0/0 (0)
Infection with neutropaenia	−/2/0 (5)	−/3/0 (8)
Infection without neutropaenia	1/0/0 (0)	4/2/0 (5)
Neuropathy	0/0/0 (0)	1/0/0 (0)
Any grade 3/4 toxicity	35 (88%)	32 (80%)
Any grade 3/4		
Non-haematological toxicity	15 (38%)	9 (23%)

**Table 4:** Clinical response to chemotherapy based on RECIST

**Arm**	**Cisplatin–docetaxel**	**Docetaxel alone**
*N*	40	39
Completed chemotherapy	38 (95%)	24 (62%)
CR	1	0
PR	17	6
CR+PR	18	6
SD	18	23
PD	4	10
NE	0	0
ORR	45%	15%
(95% confidence interval)	(29–62%)	(6–31%)

CR=complete response; NE=not evaluable; ORR=overall response rate; PD=progressive disease; PR=partial response; RECIST=Response Evaluation Criteria in Solid Tumor; SD=stable disease.

**Table 5:** Pathological results

**Arm**	**Cisplatin–docetaxel**			**Docetaxel alone**		
**c-N stage**	**N0**	**N1**	**Total**	**N0**	**N1**	**Total**
Number of cases	26	14	40	27	12	39
p-N0	17	6	23	18	4	22
p-N1	3	5	8	3	1	4
p-N2	5	2	7	5	4	9
p-N3	1	0	1	0	0	0
Not assessable	0	1	1	1	3	4

**Table 6:** Outcome at 1 year

**Arm**	**Cisplatin–docetaxel**	**Docetaxel alone**	**Total**
Number of cases	40	39	79
Alive, disease-free	31	24	55
Alive with disease	4	11	15
Dead, due to cancer	3	2	5
Dead, treatment-related	2	0	2
Dead, other causes	0	2	2

## Appendix

The following institutions and investigators participated in the trial:

Tohoku University Hospital (Takashi Kondo and Akira Sakurada), Tochigi Cancer Center (Haruhisa Matsuguma), Saitama Cancer Center (Hirohiko Akiyama), National Cancer Center Hospital East (Kanji Nagai, Junji Yoshida and Nagahiro Saijo), National Cancer Center Hospital (Hisao Asamura, Kenji Suzuki and Hideo Kunitoh), Kyorin University School of Medicine (Tomoyuki Goya and Yoshihiko Koshiishi), Tokyo Medical University (Harubumi Kato and Masahiro Tsuboi), Cancer Institute Hospital (Ken Nakagawa and Yukitoshi Satoh), Yokohama Munipical Citizen's Hospital (Koshiro Watanabe and Jun-ichi Nitadori), Niigata Cancer Center Hospital (Teruaki Koike and Yasushi Yamato), Aichi Cancer Center Hospital (Tetsuya Mitsudomi and Shoichi Mori), Osaka Prefectural Hospital Organization Osaka Medical Center for Cancer and Cardiovascular Diseases (Ken Kodama and Masahiko Higashiyama), Osaka Prefectural Hospital Organization Osaka Prefectural Medical Center for Respiratory and Allergic Disease (Mitsunori Ota), Osaka City General Hospital (Hirohito Tada and Ryoji Yamamoto), Hyogo Cancer Center (Morihito Okada, Masahiro Yoshimura and Koichiro Iwanaga), National Hospital Organization Shikoku Cancer Center (Motohiro Yamashita), National Kyushu Cancer Center (Yukito Ichinose and Koji Yamazaki), Nagasaki University School of Medicine (Takeshi Nagayasu and Tsutomu Tagawa).
